# The patient-centric development of an inclusive multi-arm multi-stage trial for Parkinson’s disease

**DOI:** 10.1093/braincomms/fcag349

**Published:** 2026-09-22

**Authors:** Marie-Louise Zeissler, Matthew Burnell, Thomas R Barber, Yoav Ben-Shlomo, Caroline S Clarke, Sally Collins, Carl Counsell, Joy Duffen, Mark J Edwards, Romy Ellis-Doyle, Cristina Gonzalez-Robles, Kate Hockey, Anna Jewell, Michael Lawton, Georgia Mills, Rebecca Petty, Dorothy Salathiel, Sue Whipps, Alan Whone, Sonia Gandhi, Camille B Carroll, Thomas Foltynie, James R Carpenter, Roger A Barker, the EJS ACT-PD Consortium, Thomas Foltynie, Camille B Carroll, Roger A Barker, James R Carpenter, Yoav Ben-Shlomo, Mark J Edwards, Alan Whone, Carl Counsell, Caroline S Clarke, Matthew Burnell, Kate Hockey, Anna Jewell, Priti Gros, Thomas Barber, Anette Schrag, Rimona S Weil, Caroline H Williams-Gray, Michele T Hu, Lynn Rochester, Paola Piccini, Henrik Zetterberg, Alastair Noyce, Michael Lawton, Ashwani Jha, Brook Huxford, Shlomi Haar Millo, K Ray Chaudhuri, Carroll Siu, Michèle Bartlett, Kuhan Pushparatnam, Daniel van Wamelen, Anthony H V Schapira, Oliver Bandmann, Simon R W Stott, George K Tofaris, Esther Sammler, Heather Mortiboys, Li Wei, Alan Wong, Susan Duty, David T Dexter, Edwin Jabbari, Stephen Mullin, Huw Morris, David Breen, Christian Lambert, Prasad Korlipara, Monty Silverdale, Kailash Bhatia, Alison J Yarnall, Raj Khengar, Helen Collins, Fleur Hudson, Rebecca Croucher, Sandra Bartolomeu-Pires, Veena Agarwal, Jennifer Allison, Jodie Forbes, Alex Edwards, Sheila Wonnacott, Dilan Athauda, Joy Duffen, Sonia Gandhi, Emily Henderson, Jen Black, Karen Matthews, Vince Greaves, Eric Deeson, Laurel Miller, Joel Handley, Helen Matthews, Kevin McFarthing, Amit Batla, Nikul Bakshi, Miriam Parry, Natasha Ratcliffe, Cheney Drew, Naveena Kapur, Anaya Navangul, Shafaq Ali, Katherine Fletcher, Claire Bale, Cristina Gonzalez-Robles, Marie-Louise Zeissler, Georgia Mills, Romy Ellis-Doyle, Sally L Collins, Rebecca Petty

**Affiliations:** Translational and Clinical Research Institute, Newcastle University, Newcastle upon Tyne NE1 7RU, UK; National Institute for Health and Care Research (NIHR) Biomedical Research Centre, Newcastle Upon Tyne NE4 5PL, UK; Faculty of Health, University of Plymouth, Plymouth, PL6 8BT, UK; Medical Research Council Clinical Trials Unit, University College London, London WC1V 6LJ, UK; National Hospital for Neurology and Neurosurgery, London WC1N 3BG, UK; Population Health Sciences, University of Bristol, Bristol BS8 2PS, UK; Research Department of Primary Care and Population Health, University College London, London NW3 2PF, UK; Translational and Clinical Research Institute, Newcastle University, Newcastle upon Tyne NE1 7RU, UK; National Institute for Health and Care Research (NIHR) Biomedical Research Centre, Newcastle Upon Tyne NE4 5PL, UK; Faculty of Health, University of Plymouth, Plymouth, PL6 8BT, UK; Institute of Applied Health Sciences, University of Aberdeen, Aberdeen AB25 2ZD, UK; Cure Parkinson’s, London W1W 6XX, UK; Institute of Psychiatry, Psychology & Neuroscience (IoPPN), King’s College London, University of London, London SE5 8AB, UK; Department of Clinical and Movement Neurosciences, UCL Queen Square Institute of Neurology, University College London, London WC1N 3BG, UK; Department of Clinical and Movement Neurosciences, UCL Queen Square Institute of Neurology, University College London, London WC1N 3BG, UK; Expert by Experience, London, UK; Expert by Experience, London, UK; Population Health Sciences, University of Bristol, Bristol BS8 2PS, UK; Department of Clinical and Movement Neurosciences, UCL Queen Square Institute of Neurology, University College London, London WC1N 3BG, UK; Faculty of Health, University of Plymouth, Plymouth, PL6 8BT, UK; Expert by Experience, London, UK; Expert by Experience, London, UK; School of Physiology, Pharmacology & Neuroscience, University of Bristol, Bristol BS8 1TD, UK; Department of Clinical and Movement Neurosciences, UCL Queen Square Institute of Neurology, University College London, London WC1N 3BG, UK; Translational and Clinical Research Institute, Newcastle University, Newcastle upon Tyne NE1 7RU, UK; National Institute for Health and Care Research (NIHR) Biomedical Research Centre, Newcastle Upon Tyne NE4 5PL, UK; Faculty of Health, University of Plymouth, Plymouth, PL6 8BT, UK; Department of Clinical and Movement Neurosciences, UCL Queen Square Institute of Neurology, University College London, London WC1N 3BG, UK; Medical Research Council Clinical Trials Unit, University College London, London WC1V 6LJ, UK; London School of Hygiene and Tropical Medicine, London WC1E 7HT, UK; Department of Clinical Neurosciences, University of Cambridge, Cambridge CB2 0QQ, UK; Cambridge Stem Cell Institute Cambridge, CB2 0AW, UK

**Keywords:** Parkinson’s disease, trial design, multi-arm multi-stage, patient and public involvement and engagement

## Abstract

There is an urgent need to speed up clinical trials for potential disease-modifying therapies that slow progression of Parkinson’s disease. The Edmond J Safra Accelerating Clinical Trials in Parkinson’s Disease (EJS ACT-PD) initiative set out to develop a patient-centric and inclusive platform trial using an innovative multi-arm, multi-stage (MAMS) design. We describe the work of the EJS ACT-PD consortium, which included over 90 experts including people with Parkinson’s disease and care partners across six working groups. There were five key challenges: developing an efficient MAMS trial that meets regulatory requirements and is acceptable to people with Parkinson’s; mitigating participant heterogeneity; reducing the risk of simultaneous arm closures; introducing different treatment regimens into a MAMS trial; and maximizing deliverability and accessibility of a MAMS trial. The final design was achieved through an iterative process and critical appraisal of data modelling exploring the impact of participant numbers per arm, trial durations, and endpoints. Every aspect of the design was informed by people with Parkinson’s disease and care partners whose advice ensured acceptability, deliverability, and accessibility. The EJS ACT-PD trial will start with one placebo and three treatment arms, each with 400 participants, with a primary endpoint at 3 years powered to detect a 30% reduction in rate of progression of MDS-Unified Parkinson’s Disease Rating Scale (MDS-UPDRS) Part I and II summed scores. Interim analyses will test for activity using the summed inverse variance weighted MDS-UPDRS Part I, II, and remote Part III scores. Ineffective arms will be closed, allowing participants to be re-randomized. Cost-effectiveness analysis is embedded. Further promising treatments will be added in future arms, subject to additional funding. To maximize accessibility and inclusivity, the trial will allow for study visits to be conducted remotely based on participant preference. National sites were selected to maximize accessibility and inclusive recruitment with core funded staff placed within experienced trial sites to support training and delivery at less experienced centres. By involving people with Parkinson’s and care partners as well as stakeholders with broad scientific expertise, we have developed an inclusive MAMS trial which captures all stages of disease with partial remote delivery and clear design rationales that will significantly speed up the testing of disease-modifying therapies in Parkinson’s disease.

## Graphical Abstract

**Graphical Abstract:**
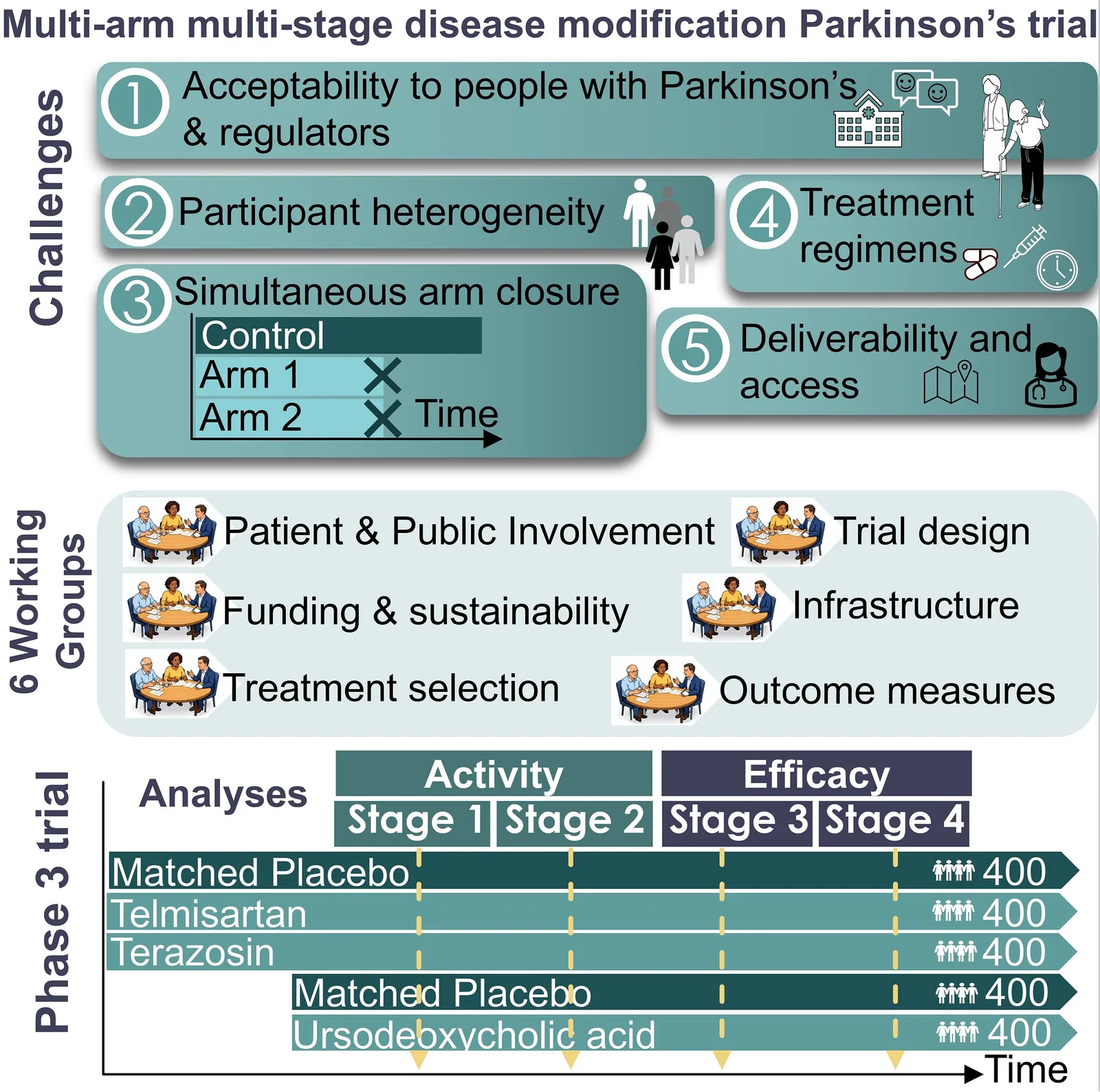

## Teaser

Zeissler *et al*. describe the development of the first multi-arm, multi-stage platform trial for Parkinson’s disease. The authors describe how insights from multiple workstreams, statistical analyses, and patient and public involvement were integrated to ensure the trial is inclusive, accessible, deliverable, and can influence policy.

## Introduction

Parkinson’s disease is one of the fastest growing neurological disorders worldwide.^1,2^ Progressive accumulation of motor and non-motor symptoms and increasing disability result in high care requirements, significantly impacting on the quality of life of affected individuals and their families. Disease-modifying therapies that delay or stop the progression of Parkinson’s disease are urgently needed to reduce current and projected economic and social impacts.^3,4^

Improved understanding of the pathogenesis of Parkinson’s disease has led to an extensive clinical and preclinical pipeline of potential disease-modifying therapies.^5-7^ However, standard double-blind randomized controlled trials (RCTs) are hindered by slow set-up times and delays to recruitment. Furthermore, progress is slow due to delays in moving from Phase 2 to Phase 3 evaluation. Reasons may include the requirement to recruit highly selected study populations, and protocols that are burdensome for sites to deliver and participants to undertake. Additionally, study populations are often not representative of the wider population of people living with Parkinson’s, impacting on the generalizability of findings.^8^ A challenge to trial design is the nature of Parkinson’s disease itself—it progresses slowly, is heterogeneous^9-13^ and symptomatic therapy may mask disease progression on clinical scales.^14-16^ These aspects are particularly problematic for trials of disease-modifying therapies.^17^

The Edmond J Safra Accelerating Clinical Trials in Parkinson’s Disease (EJS ACT-PD) initiative, launched in 2021, sought to co-develop with people with Parkinson’s (PwP) an innovative trial that would address these challenges and speed up the pace at which compounds move through the clinical trial pipeline.^17^

The benefits of a multi-arm, multi-stage (MAMS) trial design are compelling. It enables the testing of multiple treatment arms against a shared placebo group with pre-planned interim analyses, allowing for the discontinuation of arms that show no signs of activity. The adaptive platform elements accommodate the addition of new arms within the existing trial structure. Other advantages include an overall lower number of participants required to be randomized to a control group and the potential to combine traditional Phase 2 and Phase 3 evaluations within one single trial, as well as providing sustainable trial delivery infrastructure and recruitment pathways. Combined, these advantages mean that definitive answers on efficacy of new treatments can be found at a much faster pace than through a series of conventional two-arm RCTs.^18^ The MAMS trial approach has proven to provide a successful framework for progress in other disease areas,^19-23^ and, as previously described,^17,24^ offers a potential solution in Parkinson’s disease.

The aim of the EJS ACT-PD initiative was to design a trial that would provide meaningful findings, deliverable at scale in a representative population, in a way that is acceptable for participants and feasible for sites. Here, we describe the protocol development challenges and rationale for decisions made in the design of the EJS ACT-PD MAMS Phase 3 platform trial.

## Materials and methods

### The EJS ACT-PD consortium structure

**Figure 1:**
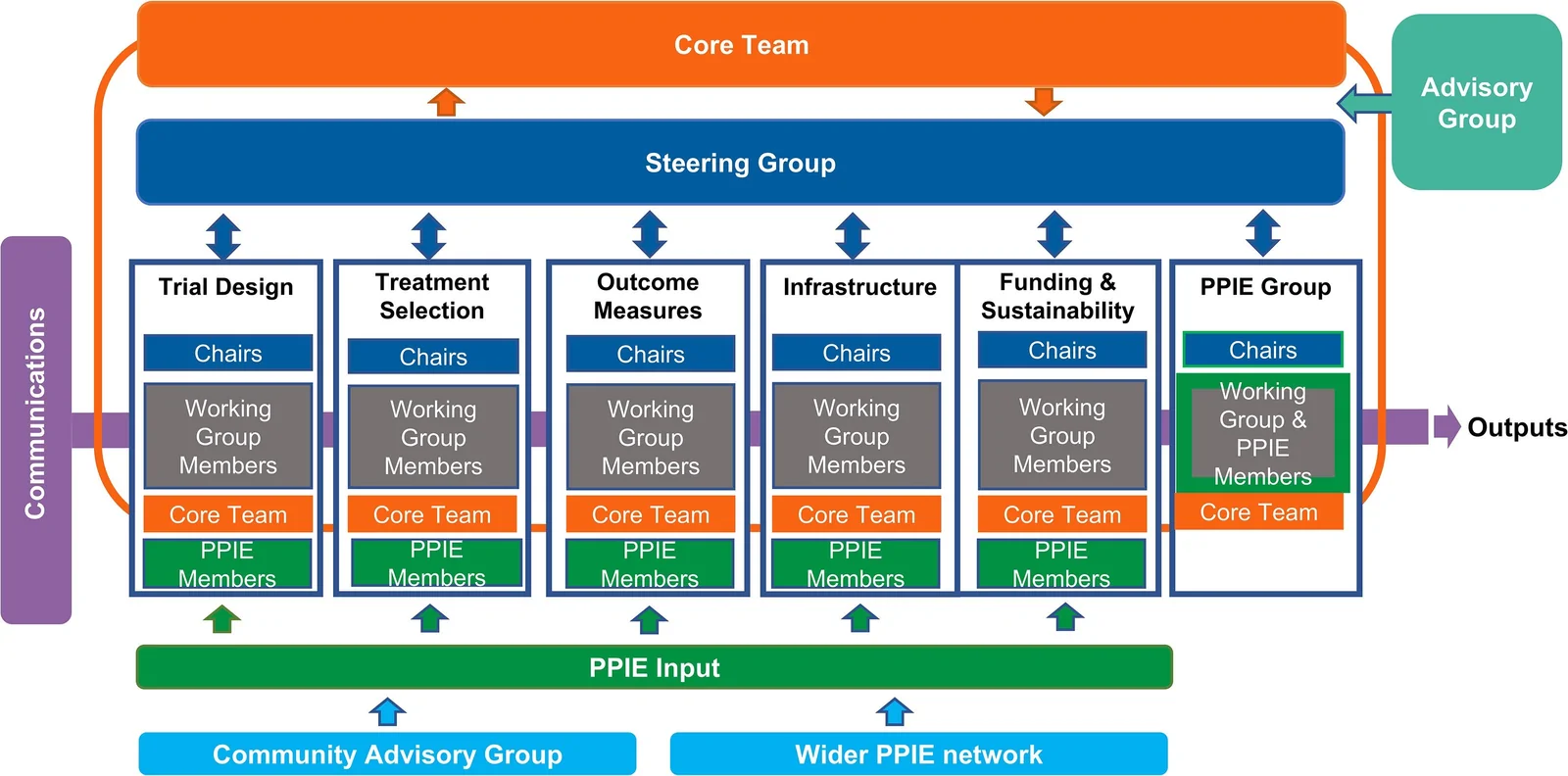
**Consortium overview.** The EJS ACT-PD consortium comprises six working groups: Treatment Selection, Outcome Measures, Funding and Sustainability, Infrastructure, Trial Design, and Patient and Public Involvement and Engagement (PPIE). Chairs of all the working groups and the initiative’s leadership form a steering group that oversees the project. An external panel of international experts in Parkinson’s trials, MAMS trials, and trial delivery form the project’s advisory group. A communications subgroup oversees the project’s communications strategy. The PPIE working group is supported by a Community Advisory Panel whose diverse membership provides targeted input into aspects concerning research equity and a Wider PPIE Network provides an additional pool of PwP and care partners for ad hoc consultation via surveys and focus groups. Abbreviation: PPIE: Patient and Public Involvement and Engagement.

The EJS ACT-PD initiative brought together over 90 experts including PwP disease and care partners (Supplement 1) across six working groups addressing different aspects of trial development (Fig. 1). The working groups are described below.

**Treatment selection**: tasked with selecting the first treatments to enter the trial. The working group developed methodology for identifying potentially suitable compounds, as well as a process for shortlisting candidates through expert and lay review.^25^

**Infrastructure**: tasked with ensuring national trial delivery capability as well as developing a feasible trial delivery strategy that promotes inclusivity and maximizes access to participation opportunities. The working group developed a process to understand and best utilize current trial delivery infrastructure and capability across the UK, informing a strategy for site selection and provision of additional infrastructure support.

**Outcome measures**: tasked with selecting a suitable outcome assessment battery and clinically meaningful primary endpoint. Primary outcome selection was informed by power and sample size modelling of outcomes with favourable clinimetric properties using established datasets. Internationally recognized core outcome sets for Parkinson’s disease trials were critically appraised considering deliverability and patient acceptability.^26,27^

**Funding and sustainability**: tasked with considering costing and funding models for the trial. The group’s membership included stakeholders such as UK Parkinson’s charities and representatives from the National Institute for Health and Care Research (NIHR) to increase transparency and strengthen collaborative working across organizations.

**Patient and public involvement and engagement (PPIE)**: tasked with providing the patient perspective in all working groups throughout the development of the trial to ensure patient-centricity. The group’s PPIE representatives were distributed across all working groups as well as coming together to discuss matters arising. Wider outreach was commissioned when required. The effectiveness and impact of the group were evaluated to continually iterate ways of working.^28^

**Trial design**: tasked with generating a master protocol for the trial, informed by the outputs of the other working groups. Its ways of working towards derivation of trial design decisions are described in detail below.

### The trial design working group

#### Composition

The trial design working group included Parkinson’s disease and MAMS trial experts, statisticians, epidemiologists, health economists, and neurologists with a special interest in Parkinson’s disease, as well as two PwP (D.S. and A.J.) and two care partners (S.W. and K.H.). The group was co-chaired by a Parkinson’s disease expert clinician and trialist (R.A.B.) and a statistician with expertise in complex innovative trial designs (J.C.).

#### Remit

The remit of the trial design working group was to generate a master protocol for a Phase 3 MAMS platform trial that would produce practice-changing results, providing inclusive opportunities for trial participation to PwP, while offering demonstrable efficiencies in terms of time and resource use (Supplement 2). The group considered:

- eligibility criteria,
- trial duration,
- power and sample size for primary and interim outcomes proposed by the outcome measures working group,
- trial operating characteristics including timepoints for planned analyses,
- plans to mitigate the impact of heterogeneity in Parkinson’s disease, aetiology and progression,
- strategies to manage delivery challenges highlighted by the infrastructure working group, and
- procedures for introducing arms with different treatment administration modalities selected by the treatment selection working group within the MAMS paradigm.

The underlying principles were that the trial should be:

1. Feasible to deliver at trial sites (low burden).
2. Acceptable to PwP/care partners (low burden and accessible).
3. Underpinned by robust modelling.
4. Inclusive (able to recruit a representative population).
5. Have a meaningful Phase 3 outcome (acceptable to regulators).
6. Efficient (identify both effective and ineffective arms as quickly as possible).

#### Decision-making process

The trial design working group employed an iterative process for reviewing statistical analyses at working group meetings supported by the review of relevant literature and drawing on the experience of other UK MAMS trials for neurodegenerative disorders^19,22^ as well as using insights from an international Delphi study as a starting point for discussions.^29^ PPIE input was integral to decision making, with PwP and care partner representation at all meetings and clear processes for consultation of wider PPIE views through the initiative’s PPIE working group, community advisory panel and wider network.^28^

#### Data analyses underpinning decision making

Detailed results of data analyses undertaken to inform decision making and reporting of associated results lie outside the scope of this manuscript which focuses on overall decision making processes in the trial design and the challenges encountered and how they are addressed. However, a brief outline of analyses and how they underpinned decision making is included in the following sections.

##### Datasets used for analyses

The primary dataset for analyses was the Critical Path Parkinson’s (CPP) Integrated Parkinson’s Database (data cut off: 7 January 2022), utilizing data from studies with >100 participants with >1 year of follow-up. Datasets from six observational studies (total *n* = 3935; male = 2519, 64%) including CamPaiGN (Cambridgeshire Parkinson’s Incidence from GP to Neurologist),^30^ ICICLE (Incidence of Cognitive Impairment in Cohorts with Longitudinal Evaluation-Parkinson’s disease),^31^ PPMI (Parkinson’s Progression Markers Initiative),^32^ OPDC (Oxford Parkinson’s Discovery Cohort),^33^ PRoBaND (Tracking Parkinson’s),^34^ PICNICS (Parkinsonism: Incidence and CogNItive heterogeneity in CambridgeShire)^35^; and six RCTs (total *n* = 2863; male = 1803, 62.9%) including FS-1 (Futility Study-1),^36^ FS-TOO,^37^ NET-PD LS1 (National Institute of Neurological Disorders and Stroke Exploratory Trials in Parkinson’s disease Long-Term Study 1),^38^ SURE PD (Study of URate Elevation in Parkinson’s Disease),^39^ STEADY PD3,^40^ and SURE-PD3,^41^ within the dataset were included in analyses.^42^ Unified Parkinson’s Disease Rating Scale (UPDRS) scores were converted into Movement Disorders Society (MDS)-UPDRS equivalents.^43^ The DATATOP (Deprenyl and Tocopherol Antioxidative Therapy of Parkinsonism) trial^44^ was excluded from the analysis as it represented a significant outlier in terms of participants’ disease progression.

##### Power calculations and trial operating characteristics

Power calculations for various combinations of the MDS-UPDRS and determination of trial operating statistics were modelled on observational study data only. Data from observational studies were more representative of the broadly inclusive study population anticipated within the Phase 3 EJS ACT-PD trial, compared with the more homogeneous participant populations of RCTs within the dataset. Analytical power calculations^45^ used mixed model parameters derived from observational CPP studies to triangulate optimal trial design conditions, exploring different effect sizes (20–50%). Participant follow-up lengths of up to 5 years were considered.^29^ Scenarios exploring different recruitment rates were factored into estimations of stage analysis timepoints for the first three arms. Calculations assumed 25% attrition after 3 years with a higher likelihood of attrition in the period following randomization (Weibull model with shape parameter = 0.75).

##### Mitigating participant heterogeneity

Analyses were undertaken to understand the impact of patient heterogeneity on disease progression as assessed by the MDS-UPDRS and thus inform discussions on how these could be accounted for in the trial design. As a result, meta-analyses based on linear random effect models were performed to explore the effect of biological sex, age, trial treatment, clinical phenotype [postural instability and gait difficulty (PIGD) or tremor dominant disease],^46,47^ time since diagnosis, ON/OFF Parkinson’s disease medication state and Hoehn and Yahr stage on clinical progression using individual parts of the MDS-UPDRS as well as combined sum scores. Factors that potentially affect disease progression or impact on primary and interim outcome measures were identified from literature review and expert elicitation. Impacts were considered by the Trial Design Working Group to inform eligibility criteria, stratification factors and planned sensitivity analyses.

#### Trial delivery considerations

Feasibility of trial delivery was informed by a survey of national site research capabilities undertaken by the infrastructure working group and PPIE working group discussions. Information was fed back to relevant working groups to devise strategies to mitigate challenges to deliverability and accessibility, considering how to maximize national trial delivery capabilities, selection of outcomes, modality of study visits, study drug dispensation, promoting participation of underserved communities, retention, platform sustainability, and pathways to impact.

## Results

**Figure 2:**
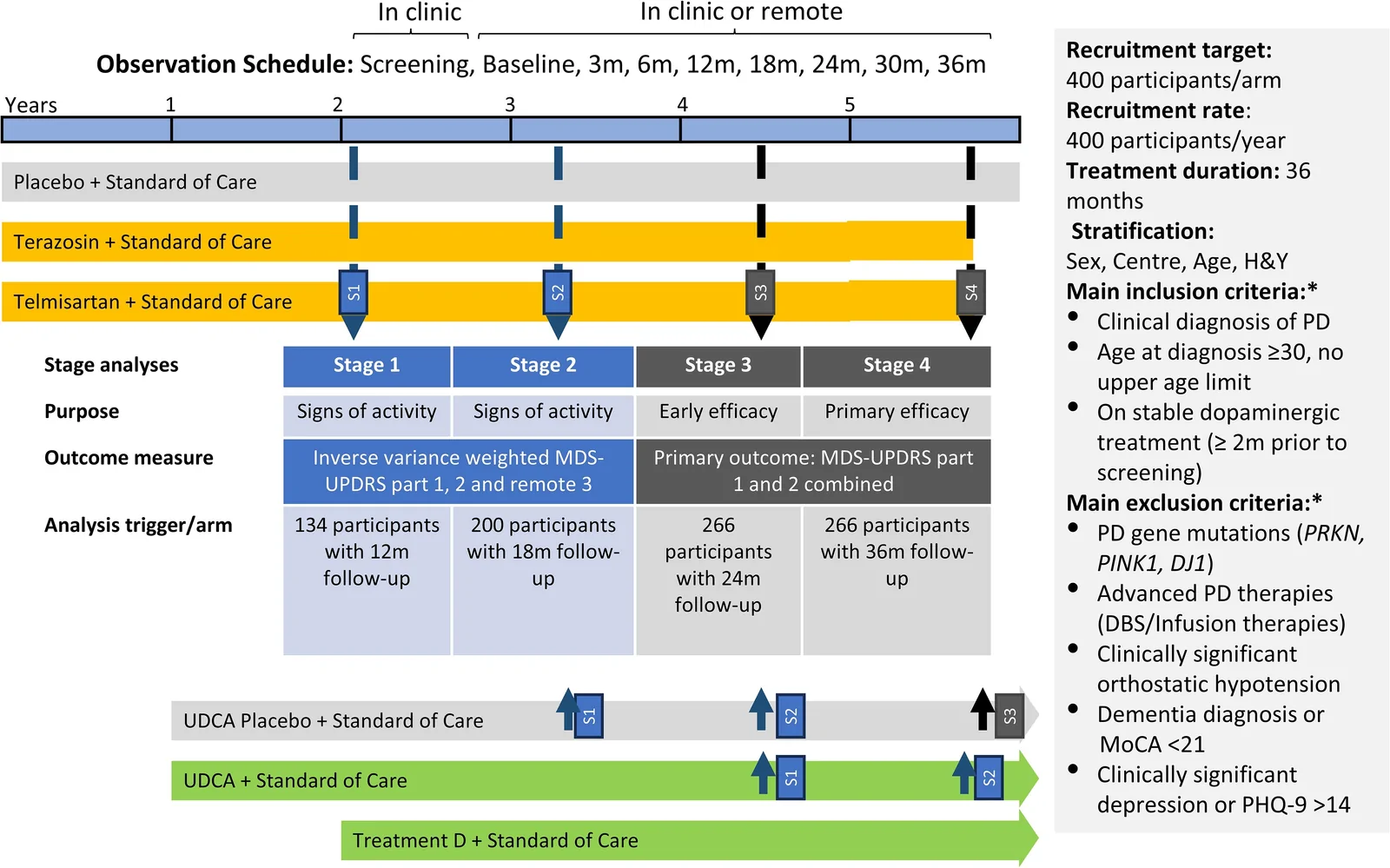
**EJS ACT-PD trial overview.** The trial will initially have three arms: two active arms and one contemporaneous placebo control arm, with a third treatment arm being introduced after 1 year. Participants will receive their allocated study treatment in addition to standard of care for a maximum of 36 months. The proposed analyses for each arm within the EJS ACT-PD trial comprise four stages, each triggered by pre-specified participant numbers reaching a follow-up timepoint per arm. Stages 1 and 2 analyses will test for activity, thus allowing for early stopping of treatments that show no sign of activity. Stage 3 will be an early test for efficacy on the primary outcome, allowing the early identification of a successful treatment. The primary efficacy analysis will be at Stage 4. Due to this follow-up milestone-based approach, overall length of the trial and analysis timepoints are dependent on recruitment rate. The *Primary Outcome* (*efficacy testing at Stages 3 and 4*) will be a change in rate of progression on the combined MDS-UPDRS Parts I + II score. The *Interim Outcome* (*activity testing at stages 1 and 2*): will be an inverse variance weighted combination of MDS-UPDRS part I, II and remotely captured part III [excluding rigidity (item 3.3) and postural stability (item 3.12)]. Abbreviations: DBS: deep brain stimulation; H&Y: Hoehn and Yahr; m: months; MoCA: Montreal Cognitive Assessment; PHQ-9: Patient Health Questionnaire; UDCA: ursodeoxycholic acid; *A comprehensive list of eligibility criteria can be found within the Supplement.

An overview of the proposed EJS ACT-PD trial design which describes the key elements including study arms, stage analyses, key eligibility criteria, and assessment schedule can be found in Fig. 2.

### Challenge 1: developing an efficient MAMS trial, capable of delivering go/no-go decisions early, that meets regulatory requirements and is acceptable to PwP

The premise of this Phase 3 trial is to demonstrate a clinically meaningful effect on disease progression to allow licensing of treatments for disease modification by regulators and adoption into clinical care. Of importance to PPIE representatives was that the trial should be as short as possible whilst still delivering results meaningful to patients and preserving inclusivity.

#### Primary outcome (efficacy testing at Stages 3 and 4)

Primary outcome selection was informed by power and sample size modelling of outcomes with favourable clinimetric properties. The positioning of the trial as Phase 3 meant the primary outcome had to be well validated, acceptable to regulators, meaningful to people living with Parkinson’s and feasible at scale.^48^ PPIE representatives were in favour of a primary outcome that captures non-motor as well as motor impact, and that would facilitate inclusivity and accessibility by being remotely deliverable.^27^ The trial’s primary outcome was therefore chosen to be a change in rate of progression on the combined MDS-UPDRS Parts I + II score. Part III was not included within the sum score of the primary outcome as it undermines clinimetric properties,^49^ this concern does not extend to the Part I + II combination.^50^ To facilitate primary outcome completion for the duration of the trial, a diverse range of assessment modalities will be permitted including via video calls, telephone calls, in-person clinic, or home visits.

#### Treatment effect size

Modelling demonstrated that a 30% reduction in the rate of progression of the primary outcome reduces the absolute risk of dementia, falls, and mortality after 10 years.^51^ This was considered a clinically meaningful and feasible change by the trial design working group and supported by the PPIE working group.

#### Interim outcome

Interim outcomes are essential for the success of a MAMS design as they will determine early stopping of ineffective treatments. Interim outcomes do not need to be fully validated surrogate endpoints, nor are they required to be a qualified Phase 3 outcome measuring a clinically meaningful aspect of health.^52^ Interim outcomes are required to be sensitive to change and correlate with or predict progression of the primary outcome. We found a novel inverse variance weighted combination of MDS-UPDRS Part I, II and remotely captured Part III ON score [excluding rigidity (item 3.3) and postural stability (item 3.12)] to have superior power in detecting changes in rates of progression earlier than the primary outcome, thereby making it an appropriate choice for an interim outcome. As with the primary outcome, the interim outcome was chosen to be deliverable remotely to facilitate participation by those who struggle to access trial sites.

#### Treatment duration

Up to 5 years of study treatment duration was explored in analytical power calculations. However, a shorter treatment duration was favoured by the PPIE working group who argued that this would be more attractive to PwP and result in a lower risk of recruitment failure, and participant withdrawals crucial for long-term retention. A 3-year treatment duration requiring 400 participants per arm (as determined by analytical power calculations) to detect a 30% reduction in rate of progression of the primary outcome was found acceptable by PPIE representatives.

#### Randomization

To minimize the number of participants being allocated to placebo, which was important to PPIE representatives, the EJS ACT-PD trial was designed with an initially equal allocation ratio across arms, similar to the approach taken in other MAMS trials for neurodegenerative diseases.^19,22^

#### Trial operating characteristics and study duration

An overview of trial operating characteristics can be found in Table 1. Operational stage analysis triggers can be found in Fig. 2.

**Table 1:** Trial operating characteristics

	Stage analyses			
	Stage 1 (activity)	Stage 2 (activity)	Stage 3 (efficacy)	Stage 4 (efficacy)
Recruitment milestone (per arm)	133 completed 12 months follow up	200 completed 18 months follow up	266 completed 24 months follow up	266 completed 36 months follow up
One-sided alpha	50%	30%	0.50%	2%
Targeted power	>90%	>90%	No target	>90%
Actual power				
Slow initial recruitment^a^	91.90%	98.30%	83.90%	97.30%
400 ppts/year	92.10%	98.20%	83.80%	97.30%
500 ppts/year	90.50%	97.70%	82.50%	97.30%
Analysis time point (estimated years after trial start)				
Slow initial recruitment^a^	2.5	4.2	5.5	6.7
400 ppts/year	2.1	3.3	4.5	5.7
500 ppts/year	1.9	2.9	4	5.1

IVW: inverse variance weighted; reduction in slope (delta) 30%; dropout assumes Weibull model with shape parameter = 0.75 and ‘survival’ at 36 months = 0.75; ^a^Recruitment assumes a slow initial rate (quadratic function) to 1.5 years, then linear recruitment until year 4 where 400 per arm is achieved; Abbreviation: ppts = participants.

For the MAMS paradigm to be feasible, the operational design needed to allow for early detection of treatment activity while minimizing the chance of stopping an arm despite the treatment being effective. Thus, interim analysis triggers were chosen so power is high (>90%). One-sided alphas were set at incrementally more stringent hurdles to reduce the likelihood of continuing an arm in the absence of a real treatment effect with one-sided alphas at 50% and 30% for Stages 1 and 2 analyses, respectively and to allow for the replacement of ineffective treatments at the earliest possible time point to maximize efficiency of the MAMS paradigm.

PPIE representatives highlighted the urgent need for new treatments to be found as quickly as possible. Stages 3 and 4 analyses will therefore be triggered at the earliest timepoint. The Stage 3 analysis, with a very small alpha criterion (probability of false positive result 0.5%), will be triggered when 266 participants have reached 24 months of follow up. The Stage 4 final efficacy analysis will include all available measurements from 400 participants per arm, triggered when 266 participants have completed 36 months of treatment.

Overall time to results is dependent on recruitment rate, which determines when follow-up milestones that trigger analyses are reached. To ensure the trial is deliverable and to allow transparent communication with prospective participants, the impact of recruitment rates on trial duration was explored (Table 1). Per-site recruitment for different scenarios and cumulative follow-up burden was assessed by the infrastructure working group which took into account average recruitment figures for Parkinson’s disease trials in the UK obtained from national trial recruitment datasets. As a result, the trial will require at least 40 sites.

### Challenge 2: mitigating participant heterogeneity

The EJS ACT-PD platform trial will recruit a broadly inclusive Parkinson’s disease population with manifest disease. This decision was guided by the findings of a multi-stakeholder Delphi study in which PwP and care partners were strongly in favour of inclusivity^29^ and supported by PPIE representatives who expressed an urgency to find new treatment options.

Considerations for mitigation strategies to reduce the impact of heterogeneity incorporated into the design (eligibility criteria, stratification factors) and the analysis (such as sensitivity analyses and identifying responders) are outlined below.

#### Eligibility criteria

Eligibility criteria represent an important step towards ensuring inclusive and representative recruitment.^8,53-55^ Exclusion criteria to minimize heterogeneity in disease progression were weighed against the risk of losing information on the safety and efficacy of treatments within excluded sub-populations.

PPIE representatives were strongly in favour of criteria that promote inclusivity, are pragmatic and require minimal additional testing. The EJS ACT-PD platform trial will therefore recruit a broadly inclusive Parkinson’s disease population with manifest disease. The final eligibility criteria represent a compromise to manage the largest contributors to heterogeneity in disease progression while preserving inclusivity where possible. Supplementary Table 1 shows the rationale for eligibility criteria within the EJS ACT-PD trial.

#### Stratification factors

Biological sex^9,12,56,57^ and age^9,57^ have been shown to significantly affect progression trajectories of PwP. The rate of change in MDS-UPDRS scores in all four parts increases significantly with Hoehn and Yahr stage,^58^ with measurement variability that has the potential to be exacerbated through varying degrees of rater experience within the participating centres.^59-61^ To ensure balance of participant heterogeneity across trial arms, we will stratify randomization by delivery site expertise, biological sex, age (below 65 or 65 and above), and Hoehn and Yahr stage (below 2.5 or 2.5 and above) using a minimization algorithm.^62^

#### Sensitivity analyses

Additional factors that may have significant impact on disease progression include a history of a REM sleep behaviour disorder,^63^ coexistent diabetes,^64^ presence of common gene mutations,^65^ levodopa equivalent daily dose (LEDD), tremor dominant versus postural instability and gait difficulty (PIGD) phenotype at baseline^66,67^ and baseline exercise levels.^68^ In addition, it is not known whether the modality of assessments (such as video call, telephone call, in-person clinic, or via home visit) has an impact on the primary outcome. These will be considered for sensitivity analyses.

#### Exploratory subgroup analyses

While inclusivity was an important factor for PwP, the identification of responder groups was highlighted as an important exploratory objective.^29^

Therefore, in arms where there is a reasonable mechanistic hypothesis for treatments to be more effective in a particular subgroup, pre-specified exploratory subgroup analyses will be considered. Importantly, to preserve statistical integrity, this will include the testing of a clear hypothesis regarding the nature and direction of expected effects, taking into consideration the expected power to detect effects and methods to adjust for multiplicity of testing. This will be supported through additional embedded sub-studies that will evaluate blood and CSF biomarkers, including genotype.

### Challenge 3: reducing the risk of simultaneous arm closures

Historically, Phase 3 success rate is low for Parkinson’s trials^24^ and therefore the risk of arm closure due to lack of therapeutic activity is high, especially at the more stringent Stage 2 analysis timepoint.

To reduce the risk of simultaneous termination of all trial arms at interim analysis stages causing temporary closure of the trial, the trial will initially have three arms: two active arms (terazosin and telmisartan) and one contemporaneous placebo control arm, with a third treatment arm [ursodeoxycholic acid (UDCA)] being introduced after 1 year.^25^ A treatment selection working group will continue to run alongside the trial to allow identification of new treatments with the aim of introducing new arms annually, subject to identifying appropriate funding support.

### Challenge 4: introducing different treatment regimens into a MAMS trial

Parkinson’s disease trials are especially vulnerable to placebo effects (where the participant’s belief in a treatment affects their response) due to the subjective nature of clinical rating scales and participant-reported outcome measures.^69,70^ The magnitude of a placebo response is variable and can be influenced by a multitude of factors (e.g. mode of administration, regimen, the look of the tablets, or a participant’s perception of the treatment’s efficacy)^71^ necessitating the inclusion of a matched, blinded placebo control arm. This is problematic for a MAMS trial design, which derives some of its efficiency through a shared placebo comparator. For the EJS ACT-PD trial this will become relevant with the introduction of the UDCA arm, which will require administration of tablets distributed across three timepoints daily, thus not matching the one tablet daily regimen of the first two arms.

The methodological challenge and risk to power resulting from introduction of non-matching placebos if retaining a combined placebo arm in MAMS trials with outcome measures susceptible to placebo responses is significant.^72^ As a result a second placebo group matching the UDCA arm will be introduced, thus fully preserving power across all comparisons. The trial will provide unique insights into placebo effects by allowing direct comparison of different placebo groups within the same trial. To further understand the placebo effect, participants will complete a participant expectations questionnaire at baseline and end of study visit to understand their beliefs regarding the effectiveness of study treatments, as well as which treatment arm they believe they have been randomized to.^73^

### Challenge 5: maximizing deliverability and accessibility of a MAMS trial

Barriers to trial delivery include both participant and site considerations: pharmacy capacity, staff capacity, burden of in-person study visits, study visit duration, burden of participation, distance from a study site, and retention challenges.

#### Maximizing national delivery capability and trial accessibility

Sites were classified into three tiers reflective of their Parkinson’s disease trial delivery experience and expertise: Tier 3 (very experienced in Parkinson’s disease trial delivery, with access to specialist investigations), Tier 2 (some experience in Parkinson’s disease trial delivery, no/limited access to specialist investigations), and Tier 1 (no experience in delivering Parkinson’s trials). Site tiers were mapped to areas with high Parkinson’s disease prevalence, percentage of the non-white population, older age, and socioeconomic deprivation ([IMD centile (Index of Multiple Deprivation)]. The site selection strategy ensured a broad geographical spread of Tier 3 sites and included coverage of areas with high ethnic diversity, deprivation and Parkinson’s disease prevalence by the 43 trial sites.^74^ To mitigate the barrier of limited staff capacity at sites, a minimum of 15, centrally funded delivery staff will be deployed at strategically selected sites across the UK to facilitate regional staff training, support assessments and develop regionally appropriate strategies to promote inclusivity.

#### Selection of outcomes

Outcome measures for the trial were selected by the outcome measures working group, to demonstrate effectiveness and cost-effectiveness of treatments aligned with regulatory requirements, while minimizing participant burden and being deliverable remotely.^26^ In addition to the MDS-UPDRS, the following outcomes will be captured in the trial:

*Clinician-reported outcomes*: Montreal Cognitive Assessment (MoCA), Levodopa-Equivalent Daily Dose (LEDD), Clinical Global Impression (Severity of Illness and Clinical Change) (CGI-S and CGI-C), modified Hoehn and Yahr Scale.

*Patient-reported outcomes*: Patient Health Questionnaire (PHQ-9), Parkinson’s Disease Questionnaire (PDQ-8), ICEpop CAPability Measure for Older people (ICECAP-O), EQ-5D-5L, Resource use questionnaire (modified CSRI and iVICQ), Study Participant Feedback Questionnaire (SPFQ), Falls questionnaire, Participant Expectation Questionnaire. The Carers Quality-of-Life questionnaire for Parkinson’s (PQoL Carers),^75^ and EQ-5D-5L will be administered to partners/spouses of trial participants as part of a partner sub-study to assess the burden and health-economic impact of trial treatments on them.

#### Modality of study visits

In-person study visits are burdensome for participants, carers, and trial sites.^76^ Remotely delivered study visits have the potential advantages of increasing accessibility and convenience of research participation. Whilst remote participation may suit individuals who live in geographically remote areas, or have limited daytime availability, some participants value in-person contact with clinical teams. PPIE contributors valued flexibility in the modality of study visit completion. A primarily remotely delivered trial also has advantages for sites as it allows for the rating of participants by individuals across organizations, thereby supporting less experienced sites and alleviating pressures on clinic space. It was therefore decided to have the option for study visits to be delivered remotely, requiring only the screening visit to be in-person (alongside a request for end of study blood sample collection). All participants will be offered annual in-person visits. A full in-person MDS-UPDRS assessment will be administered once only, at screening for all participants. For all subsequent visits, the remote MDS-UPDRS will be administered regardless of assessment location. Rater-administered assessments will be completed with participants via a video call where possible. In exceptional circumstances, certain abbreviated rater-administered assessments can be completed via telephone calls. Where participants have digital accessibility challenges, visits will be conducted in-person and where local arrangements allow, home visits by site staff will be permissible. This mix of visit modalities, whilst not expected to affect the primary outcome (MDS-UPDRS Parts 1 and 2), could affect MDS-UPDRS Part 3 ratings and therefore interim outcome for Stages 1 and 2 analyses. Previous work has highlighted that subtle changes in bradykinesia and tremor scores may be underdetected in video assessment.^77^ To mitigate this, modality of study visits will be recorded at each visit and sensitivity analyses will test the impact on outcomes (particularly MDS-UPDRS and MoCA). In addition, remote examination guides will be deployed to ensure optimal delivery.

#### Study drug dispensation

Trial site pharmacy capacity can be a barrier to trial delivery. To circumvent the need for site pharmacies to store, dispense, and distribute study medications, study medication will be delivered directly to participants’ homes via a central community pharmacy.

#### Promoting participation of under-served communities

Representative recruitment and retention of under-served communities constitutes an important aim of the EJS ACT-PD trial. These include racial and ethnic minorities, socioeconomically deprived populations, remotely living (such as rural and coastal) communities, as well as those of older age and with multi-morbidity.^8,55,78,79^ We have developed a self-referral process to increase access to the trial and monitor inclusivity across the trial in real time. All participants will be required to register their interest in the study online allowing for demographic data to be collected and tracked from registration of interest through to consent. Demographic questions were chosen to allow for comparison with UK census data and will include age, sex assigned at birth, gender, ethnicity, years in education, last employment category (for calculation of socioeconomic status using UK Office for National Statistics guidelines), and partial postcode (to monitor geographical inclusion as well as determination of the IMD, an area measure of relative deprivation^80-83^).

A recruitment and retention panel will review inclusivity of recruitment and impact on retention as well as explore innovative approaches to increase representation of under-served groups within the study. In addition, the EJS ACT-PD initiative’s current Community Advisory Panel, which includes a broad representation of people with lived experience of chronic illnesses from under-served communities,^28^ will advise on community engagement activities that help overcome barriers to inclusivity, supporting effective communication with diverse groups. A communications group will develop and execute communication strategies to support recruitment needs.

#### Promoting retention

Low levels of participant retention pose a considerable risk to trials.^55,84,85^ However, there is an existing knowledge gap on strategies to improve retention in Parkinson’s trials.^86^ PPIE representatives stressed the importance of ensuring participants are able to provide feedback of their trial experience, with a pathway to impact positively on study conduct. To facilitate this, the trial will include the SPFQ (Study Participant Feedback Questionnaire)^87^ at baseline, 18 months, and final visit. Participants can additionally provide anonymous feedback via an online portal. The SPFQ and anonymous feedback will be reviewed regularly by the trial’s Recruitment and Retention Panel to inform development of mitigation strategies for identified issues.

#### Platform sustainability and pathways to impact

The UK regulatory agency, the Medicines and Health Regulatory Agency was consulted throughout the design of the trial to ensure the trial is fit for purpose to allow for the potential licensing of therapies that are found to be effective. The integration of health economic evaluation will ensure that analyses of the relative value for money of the interventions support applications for market authorization and adoption by healthcare providers. This will help to shape future policy to ensure that effective and cost-effective therapies can be made available to clinicians and patients in a timely manner. Early and continuous engagement with potential funders, including the NIHR, was pivotal in securing a collaborative funding agreement from a consortium of funders for this first iteration of the trial and has set a framework for engaging with potential funders for future trial arms. An ongoing Funding and Commercialization working group will continue to facilitate the dialogue with regulators and explore future engagement with commercial parties who wish to enter compounds into the trial. Such engagement with industry will require flexible approaches to funding and collaboration, as well as shared risk and reward.

## Discussion

A recruitment target of 2000 participants for the first three active arms and placebo will make EJS ACT-PD the largest Parkinson’s disease trial globally. We have co-designed with patients and care partners a trial planned to be feasible, acceptable and inclusive, supported by robust data modelling, and which will be efficient and produce clinically meaningful results that can shape future healthcare policy. The EJS ACT-PD trial represents a much-needed step change in how trials for disease-modifying therapies for Parkinson’s disease are conducted, providing the opportunity to emulate other disease areas in increasing the speed of generating high-quality, practice-changing results.^20,88,89^

The planning of the trial has benefited from: access to the CPP datasets allowing analysis across >4000 individuals with Parkinson’s disease; processes that ensured the patient voice was supported and heard; early contact with regulators; and a robust framework for decision making with consultation across project workstreams.

The trial will continue to explore the challenges faced by MAMS trials in neurodegenerative diseases including how to handle placebo effects across different treatment modalities to ensure the design stays as efficient as possible. In addition, the trial will embed biosample, imaging and digital measure substudies to improve our understanding of Parkinson’s measurement and response to therapies, with the goal of improving the design of future trial arms.

The EJS ACT-PD trial will demonstrate the feasibility of long-term, large-scale, partially remote, multi-arm, Phase 3 efficacy trials for PwP disease. Making the trial broadly inclusive will enhance generalizability of findings; however, this may increase the risk of failing to find therapies that are only effective at earlier disease stages or within Parkinson’s subtypes. Pre-planned subgroup analyses will inform enrichment strategies for future trial arms.

Platform trials such as EJS ACT-PD represent a long-term investment in trial infrastructure that will enhance the efficiency of future trial arm delivery, while simultaneously supporting the development of an experienced research workforce, thereby expanding national Parkinson’s disease trial delivery capability, which will benefit Parkinson’s research beyond this individual trial. The EJS ACT-PD trial represents one of several complementary, novel platform trial approaches globally, with EJS ACT-PD being currently the only such platform within the Phase 3 setting (for a recent review we refer the reader to Fabbri *et al.*).^90^ Brought together under the collaborative umbrella of the Global Alliance for Parkinson’s Platforms initiative,^91^ they stand to deliver a true step change in the development of new treatments for Parkinson’s disease.

A key benefit of a MAMS paradigm is the ability to identify and terminate ineffective treatments early. By implication this requires a different, flexible funding model, as well as a continuous mechanism to identify and select potential new treatments. The EJS ACT-PD initiative will continue alongside the trial to support the development and sustainability of future trial adaptations, including the identification of new treatment arms and incorporation of novel outcomes. Embedded sub-studies maximize the unique opportunity afforded by the EJS ACT-PD trial to address the many gaps in understanding Parkinson’s progression and design of disease-modifying trials. Alignment with platform trials internationally^90^ will facilitate the development of shared infrastructure and harmonized datasets. By being inclusive, the trial will become more relevant to the real-world population affected by Parkinson’s disease.

## Supplementary Material

fcag349_Supplementary_Data

## Data Availability

Data sharing is not applicable to this article as no new data were created or analysed in this study. Modelling code will be available via planned further publications.

## Appendix—The EJS ACT-PD Consortium

EJS ACT-PD Consortium Membership (November 2024)

Thomas Foltynie, Camille B. Carroll, Roger A. Barker, James R. Carpenter, Yoav Ben-Shlomo, Mark J. Edwards, Alan Whone, Carl Counsell, Caroline S Clarke, Matthew Burnell, Kate Hockey, Anna Jewell, Priti Gros, Thomas Barber, Anette Schrag, Rimona S. Weil, Caroline H. Williams-Gray, Michele T. Hu, Lynn Rochester, Paola Piccini, Henrik Zetterberg, Alastair Noyce, Michael Lawton, Ashwani Jha, Brook Huxford, Shlomi Haar Millo, K. Ray Chaudhuri, Carroll Siu, Michèle Bartlett, Kuhan Pushparatnam, Daniel van Wamelen, Anthony H.V. Schapira, Oliver Bandmann, Simon R.W. Stott, George K. Tofaris, Esther Sammler, Heather Mortiboys, Li Wei, Alan Wong, Susan Duty, David T. Dexter, Edwin Jabbari, Stephen Mullin, Huw Morris, David Breen, Christian Lambert, Prasad Korlipara, Monty Silverdale, Kailash Bhatia, Alison J. Yarnall, Raj Khengar, Helen Collins, Fleur Hudson, Rebecca Croucher, Sandra Bartolomeu-Pires, Veena Agarwal, Jennifer Allison, Jodie Forbes, Alex Edwards, Sheila Wonnacott, Dilan Athauda, Joy Duffen, Sonia Gandhi, Emily Henderson, Jen Black, Karen Matthews, Vince Greaves, Eric Deeson, Laurel Miller, Joel Handley, Helen Matthews, Kevin McFarthing, Amit Batla, Nikul Bakshi, Miriam Parry, Natasha Ratcliffe, Cheney Drew, Naveena Kapur, Anaya Navangul, Shafaq Ali, Katherine Fletcher, Claire Bale, Cristina Gonzalez-Robles, Marie-Louise Zeissler, Georgia Mills, Romy Ellis-Doyle, Sally L. Collins, and Rebecca Petty.
