# Supplementary material for: The patient-centric development of an inclusive multi-arm multi-stage trial for Parkinson’s disease
Source: Brain Commun. 2026 Sep 22;8(5):fcag349. doi: 10.1093/braincomms/fcag349 (PMC13616524; doi:10.1093/braincomms/fcag349)
Supplement: Supplementary file 1 [file fcag349_supplementary_data.docx]

**Supplemental material**

1) **Trial Design Working Group Terms of Reference**

The Trial Design Working Group is responsible for generation of the master protocol for the first MAMS trial. It will:

1. Generate a menu of trial design options, including the platform design, type of adaptation, ways of including combination treatments (possibly with different mechanisms of action and therefore potentially different Phase II outcome measures), inclusion/exclusion criteria, stratification methods, placebo allocation and blinding, duration and analysis approaches, ensuring inclusivity, minimization of bias and acceptability to patients, sponsors and regulators.
2. Explore different scenarios to determine the best design type and present high level models exploiting existing datasets.
3. Consider how to reliably distinguish symptomatic effects from disease modifying effects.
4. Consider whether capable of evolving over time to maximise the chance of positive results whilst also being both practical to run and acceptable to participants.
5. Undertake modelling to understand resource requirements (time, participants and cost) of different platforms and designs.
6. Undertake robust stakeholder mapping and plan and deliver appropriate consultation events to inform trial design (for example with regulators, sponsors, funders and HRA).
7. Work with the Infrastructure Working Group to ensure that the infrastructure and processes are in place for first MAMS trial delivery, as well as future adaptations (for example stratification based on genotype)
8. Work with the Outcome Measures Working Group to develop stop/go criteria and seamless transition from phase II to phase III evaluations.
9. Develop a strategy and governance process describing the addition or termination of treatment arms.
10. The Trial Design Working Group Chair will also sit on the ACT PD Consortium Steering Committee.

The design specifically should be able to:

-produce practice-changing results, including seamless transition from phase II to phase III evaluations.

-provide options for patients across the spectrum of progressive disease.

- add in and drop treatment arms (a strategy and governance process describing the addition or termination of treatment arms should be proposed).

- run multiple arms concurrently.

- offer demonstrable efficiencies in terms of time, resource use and participants

**Time Locked Deliverables**

-Publication of a manuscript describing derivation of chosen ACT-PD Trial design.

December 2023.

-Contribution to finalized ACT-PD Protocol.

December 2023.

#### Supplementary Table 1 Eligibility criteria common to all arms

| **Criterion category** | **Eligibility criteria within the EJS ACT-PD trial** | **Rationale** |
| --- | --- | --- |
| **PD diagnosis** | Clinically established or clinically probable PD made by appropriately experienced clinicians. In the presence of any diagnostic doubt, the Movement Disorder Society criteria will be applied.[^1^](https://paperpile.com/c/0sJ18E/BQLXE)  **Exclusion:** Atypical parkinsonism, dystonic tremor, essential tremor, drug-induced parkinsonism. | Pragmatic diagnostic criteria will support recruitment at scale. The impact of potential misdiagnoses is anticipated to be lower for the manifest PD population targeted since diagnostic accuracy increases with disease duration.[^2^](https://paperpile.com/c/0sJ18E/RxUHZ) |
|  | **Exclusion:** Known carriers of PD gene mutations *PRKN, PINK1* or *DJ1*. | Mutations in listed genes lead to non-classical PD and with differential rates of disease progression. This criterion excludes a minority of PwP.[^3^](https://paperpile.com/c/0sJ18E/7TGFG) |
| **Age range** | - Age at diagnosis ≥30 - No upper age limit | The lower age limit reduces the risk of including patients with non-classical PD such as those caused by genetic mutations. Diagnosis of PD below the age of 30 is rare and therefore unlikely to impact many potential participants.  The mean age of research studies is typically lower than that seen in clinical practice. Ensuring age based eligibility criteria encourage representative recruitment is important.[^4–6^](https://paperpile.com/c/0sJ18E/AYZzK+T37sF+pCse0) |
| **PD medication** | On dopaminergic PD medication for at least 2 months prior to screening. Those on monoamine oxidase-B inhibitors only are excluded. | Stable dopaminergic PD medication is required to avoid the positive impact of starting PD medication impacting on rate of change in primary outcome thereby biassing results. The two months timeframe will allow dosing of PD medication to be optimised. UK practice guidelines support early initiation of dopamine replacement therapies,[^7^](https://paperpile.com/c/0sJ18E/GHX6x) therefore this restriction will have negligible effect on recruitment. Levodopa equivalent daily dose (LEDD), will be recorded for each study visit. |
|  | **Exclusion:** Currently taking or on waiting list for advanced therapies | Those on advanced therapies were considered less likely to be able to comply with study requirements for the duration of the study thereby impacting retention within a long term, largely remote trial. |
|  | **Exclusion:** Participants who have had deep brain stimulation (DBS) or are on a waiting list for brain surgery including deep brain stimulation. | *Risks to safety reporting:*  Brain surgery including DBS patients were considered at higher risk for surgical complications (e.g. during battery changes) thereby increasing SAE/AE reporting.[^8^](https://paperpile.com/c/0sJ18E/V7LAD)  *Risks to primary outcome and stage analyses:*  Similar to other symptomatic therapies, DBS impacts MDS-UPDRS measurements[^8^](https://paperpile.com/c/0sJ18E/V7LAD) and success of symptom control may depend on amplitude/pulse width frequency settings.[^9,10^](https://paperpile.com/c/0sJ18E/jAjmN+Q93JS) There is no recognised way to adjust for this in analyses. |
| **Inability to comply with study requirements** | **Exclusion:**   - Clinical diagnosis of dementia or MoCA <21 - Diagnosis of clinically significant depression, >14 on PHQ-9 or record of suicidal ideation within one year prior to screening and evidenced by answering "yes" to questions 4 or 5 on the Columbia-Suicide Severity Rating Scale (C-SSRS) | Those with dementia[^11^](https://paperpile.com/c/0sJ18E/6PXTt) or depression[^12^](https://paperpile.com/c/0sJ18E/fNP9X) were considered less likely to be able to comply with study requirements for the duration of the study thereby impacting retention within a long term, largely remotely delivered trial. |
| **Exposure to IMP** | A 6 month washout period prior to screening must be observed if  ➢ Patients are currently in another interventional trial  ➢ Were exposed to any IMP within an interventional trial including exposure to any currently recruiting IMPs within EJS ACT-PD.  ➢ EJS ACT-PD participants are re-enrolling (unless the treatment was stopped for lack of activity, in which case only a 6 week wash-out applies). | Of primary concern was the preservation of a true baseline for trial outcomes. Trial re-entry rule considerations included the PPIE concern that participants recruited close to planned interim analyses may have only just started treatment allocation to an arm that is discontinued. To ensure the integrity of safety reporting within the EJS ACT-PD trial a shorter, 6 week wash-out period applies for re-enrolling EJS ACT-PD participants, provided they still meet eligibility criteria). |
| **Women of child bearing potential** | ➢ Currently not pregnant, breastfeeding or intending to become pregnant during the study or within 70 days after final dose of study drug. A negative pregnancy test for women of childbearing potential is required at screening.  ➢ Women of childbearing potential who are participating or have partners that are participating in the trial must take appropriate contraceptives. | To minimise risk of harm to women of childbearing potential and infants. |
| **Co-morbidities** | **Exclusions:**  ➢ Any concurrent medical condition, abnormal laboratory tests, progressive neurological disorder (other than PD) or uncontrolled, clinically significant systemic disease that, in the opinion of the Investigator, could cause study participation to be detrimental to the participant.  ➢ Individuals with a confirmed diagnosis of cancer that requires active management and/or in the view of the local team, the diagnosis and/ or its treatment may compromise their ability to remain participating in the trial for 36 months or tolerate any of the active treatments..  ➢ Participants with hepatobiliary disorders or abnormal liver function tests (ALT or AST >2x the upper limit of normal; serum bilirubin >1.5x ULN (except for participants with Gilbert’s disease, for whom the upper limit of serum bilirubin is 51.3 µmol/l) at the screening visit.  ➢ Participants with either of the following:  ○ Sitting Systolic Blood Pressure (SBP) less than 100 mmHg or sitting Diastolic Blood Pressure (DBP) less than 50 mmHg, irrespective of symptoms  ○ Orthostatic hypotension (manifest as: Decrease in BP > 20 mmHg systolic or > 10 mmHg diastolic on supine to standing, associated with clinical symptoms; Decrease in BP >30mmHg systolic and/or BP >15 mmHg diastolic on supine to standing regardless of symptoms; If the lowest BP on standing is less than 100 mmHg or lowest diastolic on standing is less than 50 mmHg) | To minimise risk of harm to participants and loss of follow-up. |
| **Substance abuse** | A history of alcohol/drug abuse/dependence within the past 3 years prior to screening visit. | To minimise risk of harm to participants, ensure participants are able to comply with study requirements and preserve the integrity of outcome measurements including safety reporting. |
| **Treatment specific eligibility** | Meeting eligibility for at least one active treatment arm. |  |

1.	[Postuma RB, Berg D, Stern M, et al. MDS clinical diagnostic criteria for Parkinson’s disease. *Mov Disord*. 2015;30(12):1591-1601.](http://paperpile.com/b/0sJ18E/BQLXE)

2.	[Postuma RB, Poewe W, Litvan I, et al. Validation of the MDS clinical diagnostic criteria for Parkinson’s disease. *Mov Disord*. 2018;33(10):1601-1608.](http://paperpile.com/b/0sJ18E/RxUHZ)

3.	[Cookson MR. Parkinsonism due to mutations in PINK1, parkin, and DJ-1 and oxidative stress and mitochondrial pathways. *Cold Spring Harb Perspect Med*. 2012;2(9):a009415.](http://paperpile.com/b/0sJ18E/7TGFG)

4.	[Vaswani PA, Tropea TF, Dahodwala N. Overcoming Barriers to Parkinson Disease Trial Participation: Increasing Diversity and Novel Designs for Recruitment and Retention. *Neurotherapeutics*. 2020;17(4):1724-1735.](http://paperpile.com/b/0sJ18E/AYZzK)

5.	[Hart RG, Pearce LA, Ravina BM, Yaltho TC, Marler JR. Neuroprotection trials in Parkinson’s disease: systematic review. *Mov Disord*. 2009;24(5):647-654.](http://paperpile.com/b/0sJ18E/T37sF)

6.	[McGhee DJM, Ritchie CW, Zajicek JP, Counsell CE. A review of clinical trial designs used to detect a disease-modifying effect of drug therapy in Alzheimer’s disease and Parkinson's disease. *BMC Neurol*. 2016;16:92.](http://paperpile.com/b/0sJ18E/pCse0)

7.	[National Institute for Health and Care Excellence. Parkinson’s disease in adults NICE guideline [NG71]. July 2017. Accessed July 2024.](http://paperpile.com/b/0sJ18E/GHX6x) <https://www.nice.org.uk/guidance/ng71/chapter/Recommendations#pharmacological-management-of-motor-symptoms>

8.	[Petersen JJ, Kamp CB, Faltermeier P, et al. Deep brain stimulation for Parkinson’s disease: systematic review with meta-analysis and trial sequential analysis. *BMJ Med*. 2024;3(1):e000705.](http://paperpile.com/b/0sJ18E/V7LAD)

9.	[Moro E, Esselink RJA, Xie J, Hommel M, Benabid AL, Pollak P. The impact on Parkinson’s disease of electrical parameter settings in STN stimulation. *Neurology*. 2002;59(5):706-713.](http://paperpile.com/b/0sJ18E/jAjmN)

10.	[Koss AM, Alterman RL, Tagliati M, Shils JL. Calculating total electrical energy delivered by deep brain stimulation systems. *Ann Neurol*. 2005;58(1):168; author reply 168-169.](http://paperpile.com/b/0sJ18E/Q93JS)

11.	[Dalrymple-Alford JC, MacAskill MR, Nakas CT, et al. The MoCA: well-suited screen for cognitive impairment in Parkinson disease. *Neurology*. 2010;75(19):1717-1725.](http://paperpile.com/b/0sJ18E/6PXTt)

12.	[Kroenke K, Spitzer RL, Williams JB. The PHQ-9: validity of a brief depression severity measure. *J Gen Intern Med*. 2001;16(9):606-613.](http://paperpile.com/b/0sJ18E/fNP9X)
